# Public health-focused use of COVID-19 rapid antigen and PCR tests

**DOI:** 10.1038/s41598-023-50533-2

**Published:** 2024-01-16

**Authors:** Yonatan Woodbridge, Yair Goldberg, Sharon Amit, Naama M. Kopelman, Micha Mandel, Amit Huppert

**Affiliations:** 1 The Gertner Institute for Epidemiology and Health Policy Research, Sheba Medical Center, Ramat Gan, Israel; 2 Department of Computer Science, Holon Institute of Technology, Holon, Israel; 3 The Faculty of Data and Decision Sciences, Technion-Israel Institute of Technology, Haifa, Israel; 4 Clinical Microbiology, Sheba Medical Center, Ramat Gan, Israel; 5 Statistics and Data Science, The Hebrew University of Jerusalem, Jerusalem, Israel; 6 Faculty of Medicine, Tel Aviv University, Tel Aviv, Israel

**Keywords:** Computational biology and bioinformatics, Diseases

## Abstract

During the Covid-19 pandemic, accurate PCR tests were augmented by the cheap, rapid, and logistically convenient, yet less sensitive antigen tests. In Israel, a testing policy shift was implemented due to limited availability of PCR tests during the Omicron surge. Thus, both PCR and antigen tests were used, as this was the only alternative for mass testing and surveillance at the time. Yet, evidence-based surveillance requires a robust understanding of the expected consequences of changing the testing policy. Using 41,065 paired tests performed by trained staff between January and April 2022 in Israel, we estimate how the sensitivity of antigen tests changes as a function of Ct value and other key covariates. The results reveal a logarithmic relationship between antigen detection probability and viral load, as quantified by Ct-values of the PCR tests. Further analysis shows a statistically significant association with an odds ratio of approximately 0.76 with each unit of Ct-value. The analysis suggests that in spite of their compromised sensitivity, antigen tests are a natural solution for routine use, while PCR tests should be considered in situations where a false negative result could have serious consequences. These findings are the foundations of policies that will utilize the strengths of the different tests, and achieve enhanced hybrid surveillance.

## Introduction

In order to make informed decisions during pandemics, there is a need for good and reliable data. One important measure is the number of people infected daily. The accuracy of such information depends on the sensitivity and specificity of the detection test. Two types of Covid-19 tests are commonly in use—nucleic-acid amplification tests (NAAT), most commonly the polymerase chain reaction (PCR), intended to detect numerous genes within the viral genetic material (RNA), and the antigen tests (AG test), which detect viral proteins. While the AG test is highly specific, it is less sensitive than NAAT^1–5^. On the other hand, it is rapid, does not require skilled personnel, dedicated equipment or infrastructure. Further, it is relatively cheap and can be performed in almost every setup by medical teams or by untrained patients and their families. Thus, the daily numbers of AG tests that can be conducted and analyzed are almost unlimited. The resources to perform NAAT and its turnaround time make it less accessible and limits its amounts, and it is highly dependent on professional labs’ capacities and local monetary constraints. The very high sensitivity of NAAT may also enhance the discrepancy between a positive result and an “infectious status”, e.g. by detecting viral leftovers long after recovery^6^, overestimating the actual number of potentially infectious patients^3^. Thus, there is a tradeoff between test specificity, sensitivity, logistics, turnaround time (e.g., how fast results are obtained; sending the swabs to the laboratories) and cost, as manifested by the difference between NAAT and AG tests. A typical example is the contribution of AG tests to the containment of the pandemic in Hong Kong^7^, by speeding up the detection and isolation of infected persons; the considerably slower PCR test and its associated complex logistic can cause difficulties in curtailing outbreaks by methods of rapid detection and isolation. The pros and cons of each testing methodology call for policy assessment and for the design of optimal testing strategies^8,9^, which are highly dependent on the quantitative differences between the two tests.

A drastic shift in testing policy was seen in Israel during the Omicron outbreak. Over 14 million Covid-19 PCR tests were documented between February 2020 (at the beginning of the pandemic in Israel) and July 2021, while almost no infections were documented by AG test^10^. During this period most AG tests were used for gating in schools, restaurants, cultural or sports events, while the PCR was the backbone of the Israeli surveillance system. The introduction of the highly transmissible Omicron variant to Israel led to a massive outbreak in December 2021 that was characterized by an extremely sharp increase in cases, from about 6600 confirmed cases on January 1st, reaching tenfold of cases at the peak, on January 29th. Due to limitations in PCR testing capacities (the Israeli system could conduct ~ 200,000 daily PCR tests out of a population of ~ 9.5 million), a big shift from PCR to AG Covid testing occurred, and by March 30, 2022, over 30% of the documented tests were based on AG. The shift was implemented to ensure the following objectives were met: (i) maintaining ongoing surveillance, (ii) prompt identification of new cases, facilitating the timely isolation and quarantine of infected individuals, (iii) efficient administration of antiviral medication, thereby reducing the likelihood of severe illness.

To evaluate the AG test’s performance, it is not sufficient to examine only the overall analytical sensitivity and specificity rates. Several other factors contribute to the chance of testing positive, such as the time from disease onset or viral load (VL)^11,12^. These two highly correlated factors are associated with patients’ level of infectiousness^13,14^. Thus, despite having a lower sensitivity rate, the AG test might still be effective in tracking infectious patients. Indeed, studies showed increased AG detection rates given higher VL^12^. Continuing this research line, we examine the relation between VL and AG detection rates using Israel’s national AG and PCR tests database, focusing on tests that were performed by trained staff between January and April 2022. As in many other studies^13,14^, we use the cycle threshold (Ct) value of quantitative real-time PCR as a proxy for VL. The Ct is inversely correlated to the VL. Thus, the lower the Ct, the higher the VL, and the patient is potentially more infectious.

Considering the PCR test as a gold standard, we aim to estimate the ‘real life’ probability of obtaining a positive AG test, given a positive PCR, and to explore how it is affected by the Ct value and other covariates. Given the distribution of Ct values in the population, this enables the estimation of the infection proportion that would have been undetected under various policies, e.g., only AG tests, or some combination of AG and PCR tests. Answers to these questions may enable efficient future usage of testing methodologies and resources in different pandemic and epidemic settings and stages.

## Results

**Figure 1:**
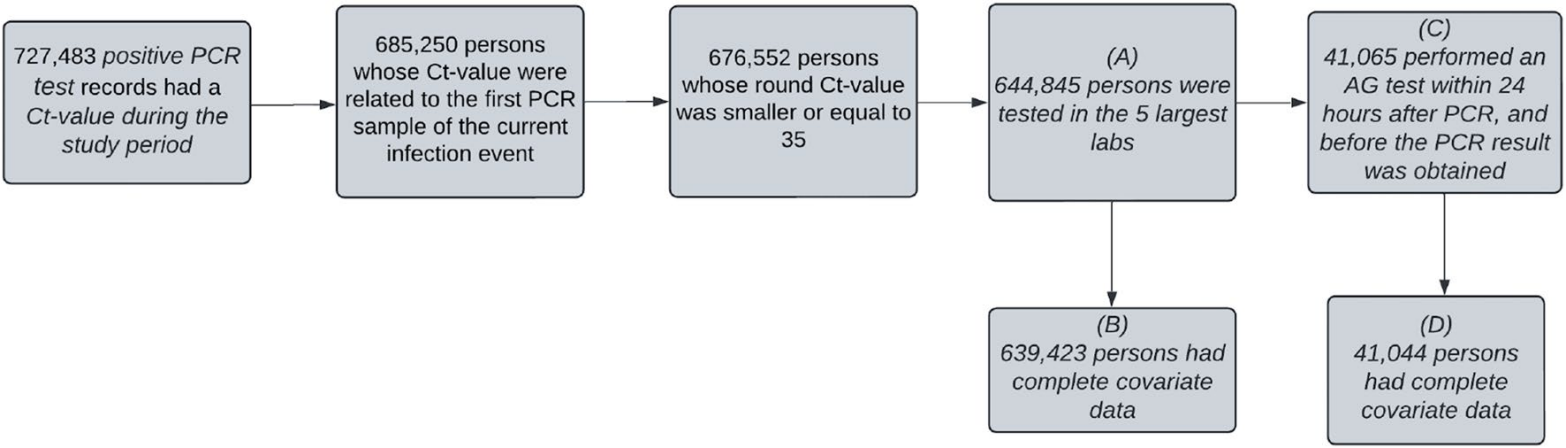
Flowchart of individuals in the study, depicting the process of data filtering.

Figure 1 illustrates the major steps performed in the data preprocessing, starting from a table containing 727,483 N-gene Ct-value records. For each person, we defined an infection event if there was either no previous infection or at least 90 days had passed from the previous infection (i.e., from the first positive PCR test in that interval)^15^. In the second step, only Ct records related to the patient’s first positive PCR test of the first infection event during the study period were included. Filtering out extreme Ct-values (rounded values above 35), the resulting data table contained 685,250 Ct-value records, each corresponding to a unique individual. Out of 676,552 persons with a confirmed infection case during the study period whose N-gene rounded Ct-value was 35 or below, 644,845 (~ 95.3%) were tested in the five largest labs. These records also included 41,065 individuals who performed an additional AG test within 24 h after the PCR test, and before the PCR test result was obtained.

Table 1 presents the distribution of different covariates in the overall population (Box B, Fig. 1) and compares it to the study subpopulation (Box D, Fig. 1). Most of the tests (more than 85%) were done during January and February which represents the peak period of the Omicron surge in Israel^10^. About 40% of the study subpopulation is of the age group 16–39 while elderly people aged 60 or more account for less than 6%. The age distribution differs from that of the overall population where individuals aged 16–39 comprise less than 35% and elderly individuals account for 15% of the PCR samples. Interestingly, there are more women than men in both the overall and the study population (60% vs 40%). There are some differences in the distributions of vaccination status and lab between the overall population and the study population. The rightmost column of Table 1 presents the percentages of positive AG tests in the different groups, which vary from 58 to 79%, in all groups except tests performed during March and April showing a larger probability (85–87.5%) of a positive AG test. There is quite a clear association between a positive AG result and time, where a larger proportion of positive results are obtained in later months. Other interesting findings are a smaller proportion of positive AG tests in children 0–2 and 3–4 compared to adults, a larger positive proportion in men compared to women, and differences among the labs.

**Table 1:** The number of PCR samples in the data with and without an additional AG test, and the AG detection rates within each category stratified by different variables.

Variable	Category	All PCR tests n (prop)	PCR tests with AG n (prop)	% positive AG
Month	JAN	420,825 (65.8%)	27,372 (66.7%)	68.10
	FEB	138,083 (21.6%)	10,772 (26.2%)	75.40
	MAR	44,625 (7%)	1954 (4.8%)	84.90
	APR	35,890 (5.6%)	946 (2.3%)	87.40
Age	0–2	12,711 (2%)	721 (1.8%)	57.60
	3–4	14,845 (2.3%)	1074 (2.6%)	62.90
	5–11	82,061 (12.8%)	5746 (14%)	70.70
	12–15	43,148 (6.7%)	2777 (6.8%)	74
	16–39	218,036 (34.1%)	17,217 (41.9%)	71.50
	40–59	172,755 (27%)	11,196 (27.3%)	71.30
	60 +	95,867 (15%)	2313 (5.6%)	75.20
Sex	F	394,625 (61.7%)	24,022 (58.5%)	68.80
	M	244,798 (38.3%)	17,022 (41.5%)	74.60
Vaccination status	Unvaccinated	140,629 (22%)	7961 (19.4%)	68.30
	1-dose	17,996 (2.8%)	1140 (2.8%)	69.20
	2-dose	93,232 (14.6%)	6510 (15.9%)	73.60
	3-dose	307,536 (48.1%)	21,823 (53.2%)	71.90
	4-dose	32,868 (5.1%)	831 (2%)	78.50
	Recovered	23,526 (3.7%)	1245 (3%)	68.10
	Recovered + vaccinated	23,636 (3.7%)	1534 (3.7%)	66.70
Lab	Lab 1	203,146 (31.8%)	9982 (24.3%)	66.90
	Lab 2	135,081 (21.1%)	11,763 (28.7%)	74.60
	Lab 3	123,116 (19.3%)	8100 (19.7%)	68.30
	Lab 4	100,510 (15.7%)	8064 (19.6%)	76.30
	Lab 5	77,570 (12.1%)	3135 (7.6%)	66.90

Figure S1 in the supplementary material compares the Ct distribution of those who underwent both PCR and AG tests to the entire study population which underwent PCR tests, regardless of whether or not an AG test was performed within 24 h. The Ct values in the general population tend to be somewhat lower (mean 26.25 compared to 26.09), but the difference between the distributions is quite small with the mean difference far below one Ct cycle.

**Figure 2:**
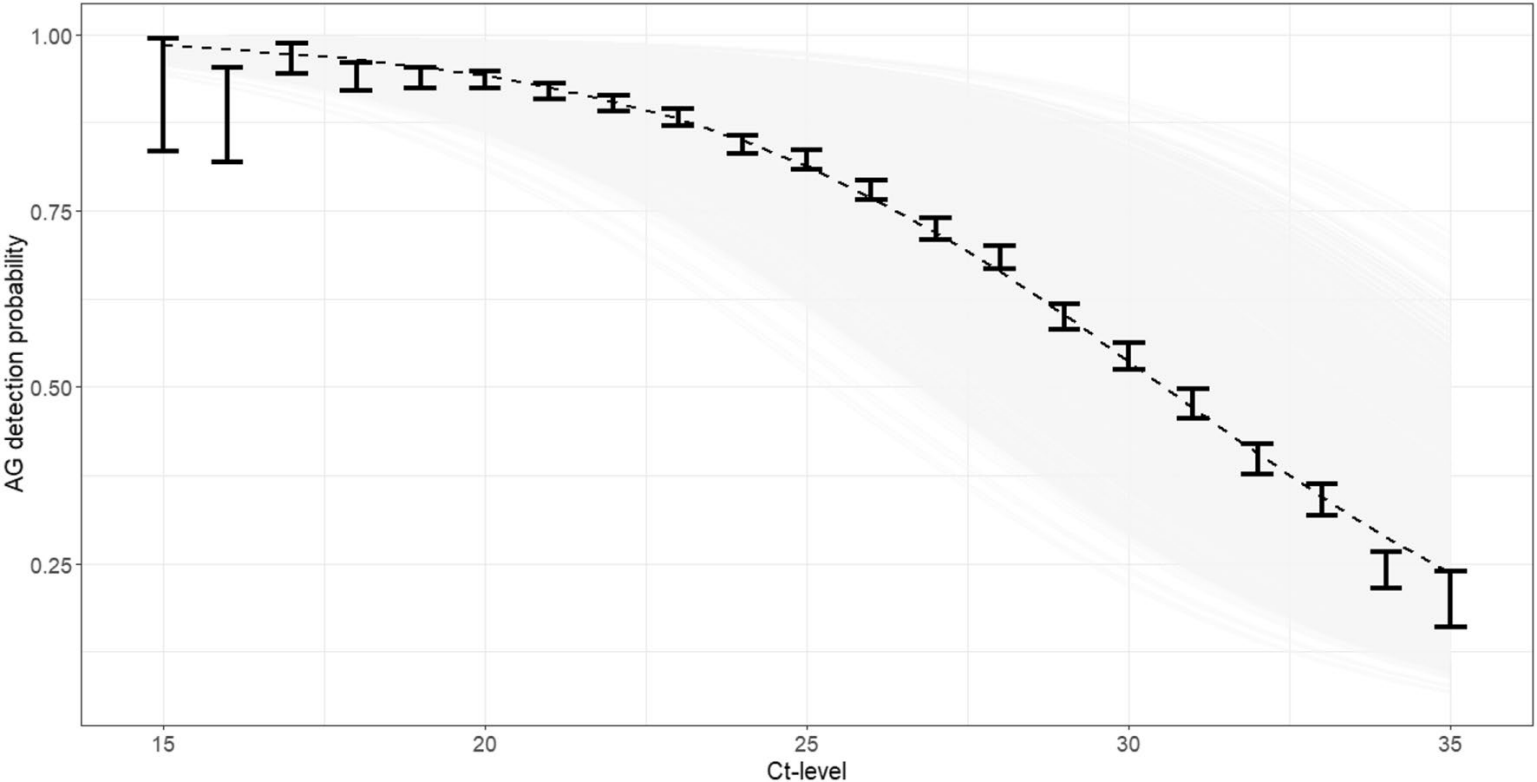
The AG detection probability vs Ct-levels for gene N, measured by 5 labs between January and April 2022. Vertical lines are proportion and 95% confidence interval calculated separately for each Ct integer value. The dashed line is a univariate logistic regression of Ct value on a positive AG test. The gray lines are the estimated multivariate logistic curves for each individual in the study population. The data that was used to create this graph is described in Box C of Fig. 1 (Box D for the gray curves).

Figure 2 illustrates the relation between Ct-level and AG detection probability. The vertical bars represent 95% confidence intervals around the proportions in each Ct category (integer values). The dashed line represents an estimated univariate logistic model over the Ct-values. As can be seen from the graph, the empirical AG detection probability closely approximates a logistic curve. This graph asserts that a decreased Ct level (i.e., increased viral load) renders AG detection more probable in a nonlinear fashion. Note that the detection probability for Ct-values less than 23 is very high. Figure S2 repeats the analysis in Fig. 2, stratified by the different covariates, while Fig. S3 presents stratification by lab. It shows a new, more detailed look at the results provided in the rightmost column of Table 1. The differences in AG detection probabilities between months, sex, and age groups are clearly observed. One can use these plots to evaluate detection rates in worst/best case scenarios. For instance, in cases where samples were taken in January vs March 2022 (see Fig. S2 for differences in detection rates).

Table 2 reports the results of a multivariate logistic regression on a positive AG test, with Ct as a continuous exposure, adjusting for all covariates as mentioned in the Methods section. The Ct coefficient is − 0.27, translating to an odds ratio estimate of about 0.76. This is quite a large effect for a continuous variable ranging from 15 to 35. There is a significant effect for children aged 0–2 and 3–4, showing smaller probabilities to be detected by AG (OR = 0.63 and 0.70, respectively). Males have a significantly larger probability of being confirmed by an AG test, with an odds ratio of 1.32. In addition, there are clear differences between the labs, possibly due to differences in assays, or different methodological standards. Interestingly, vaccination seems to be slightly positively correlated with the probability of AG detection. Finally, the probability of detection increases almost monotonically with time (i.e., calendar date), even after correcting for the Ct level. Mass AG testing in Israel started in January; at that time many teams were less experienced. Thus, accumulated experience in swab sample collection and rapid tests conduction, and possibly an improved sensitivity of AG kits, might all explain the increase in AG detection over time. The shaded area in Fig. 2 displays the variability in AG detection probabilities among individuals. It was obtained by estimating and plotting the logistic curves of all individuals in the study group, using the multivariate logistic regression results.

**Table 2:** Multivariate logistic regression results.

Variable	Category	Coefficient (95% CI)
Intercept		7.34* (7.13, 7.56)
Ct-value		− 0.27* (− 0.28, − 0.26)
Age	0–2	− 0.47* (− 0.66, − 0.28)
	3–4	− 0.35* (− 0.51, − 0.18)
	5–11	− 0.02 (− 0.12, 0.07)
	12–15	0.07 (− 0.04, 0.18)
	16–39	Ref
	40–59	− 0.05 (− 0.11, 0.01)
	60 +	− 0.11 (− 0.23, 0.02)
Sex	F	Ref
	M	0.28* (0.23, 0.33)
Lab	Lab 1	Ref
	Lab 2	0.43* (0.37, 0.5)
	Lab 3	0.34* (0.27, 0.41)
	Lab 4	0.7* (0.62, 0.77)
	Lab 5	0.03 (− 0.07, 0.13)
Vaccination status	Unvaccinated	Ref
	1-dose	0.06 (− 0.1, 0.21)
	2-dose	0.11* (0.02, 0.21)
	3-dose	0.08 (− 0.01, 0.17)
	4-dose	0.22 (− 0.01, 0.45)
	Recovered	− 0.04 (− 0.18, 0.11)
	Recovered + vaccinated	0.04 (− 0.11, 0.18)
Time period (days from 1/1/2022)	0–13	Ref
	14–27	0.38* (0.31, 0.45)
	28–41	0.6* (0.53, 0.68)
	42–55	0.88* (0.77, 0.99)
	56–69	1.24* (1.04, 1.45)
	70–83	1.34* (1.14, 1.56)
	84–97	1.15* (0.95, 1.36)
	98–111	1.51* (1.19, 1.86)
	112–119	2.22* (1.45, 3.15)

The data that was used to create this graph is described in Box D of Fig. 1.

(*) ≤ 0.05 significance.

Finally, we used our model to estimate the expected number of undetected individuals in the hypothetical case that only AG tests were performed. This was done by applying the results of the multivariate model to the overall population who have full covariate data (Fig. 1, Box B). Using only AG tests would result in 70% detection rates. In other words, out of the 639,423 infections that were confirmed by PCR, only 450,438 positive tests would be obtained if the PCR test were replaced by AG tests, thus missing about 188,985 individuals in this period. A similar analysis predicts an increase from only 62 to 87% detection rate on days 0–13 (January) and days 98–111 (April) from study follow up, respectively.

## Discussion

AG tests offer major advantages over conventional PCR tests, such as low cost, rapid results, minimal equipment, infrastructure and training requirements, and, most importantly, negligible logistical efforts. Indeed, early in the pandemic cheap and rapid AG tests were suggested as a good alternative to PCR for monitoring and surveillance purposes^16^. For AG tests to be optimally used in surveillance, it is important to estimate the accuracy of the tests under different scenarios and regimes. Nevertheless, previous studies were conducted on a small scale and on selected populations.

This study is the first large-scale analysis of real-life data comparing the relative sensitivity of PCR and AG. Using a unique Israeli dataset, we constructed a database of individuals who tested positive for COVID-19 by PCR and had same-day professionally-performed AG tests. This data was generated during the Omicron BA.1/BA.2 outbreak in Israel, where testing demands exceeded the national PCR laboratory capacities, thus leading to a shift from a PCR-dominated regime to a combination of PCR and AG tests.

Using 41,065 paired tests from this period, we constructed statistical models, and estimated how the sensitivity of AG tests changes as a function of Ct value and other key covariates. The analysis showed that the probability of a positive AG test was highly correlated with the Ct value (Fig. 2) and ranged from ~ 25% for high Ct values (~ 35, low viral load) to almost 100% when the Ct value was low (< 20, high viral load). For the most frequent Ct values around 25, the sensitivity was about 80%.

Our findings support AG tests as a good tool overall for surveillance. More so, the models corroborated that Ct value is a good predictor of the probability of detection by an AG test at the population level. Nonetheless, the multivariate analysis suggested that other covariates additionally affect the probability of detection at the individual level, leading to large variability among different populations and other explanatory variables. For instance, similar to previous reports^17^, we found a lower sensitivity of the AG test among young children and females (Table 2). We suggest that the lower sensitivity might be partially explained by the fact that, in our pairing, we included in the study only cases that included both a positive PCR test and an AG test. If the probability of a positive AG test depends on the amount of viruses in the sample combined with possible lower virus shedding and/or more gentle swabbing of infants, this can lead to reduced relative detection by AG tests.

Based on this study, AG tests are a plausible solution for routine use. Nonetheless, high sensitivity is crucial under certain scenarios, and PCR tests should be considered in situations where a false negative result could have serious consequences, either in addition to, or instead of, an AG test. Such situations include testing individuals at risk for developing a severe disease, and who may benefit from antiviral treatment, or testing travelers to reduce the risk of either exportation or importation of high risk pathogens. In addition, PCR tests may prove to be beneficial for surveillance of healthcare workers or residents of nursing homes, and in other situations where spreading among high-risk individuals is more likely. One might hope that testing policies will soon be tailored in a personalized fashion, based on known risk factors such as age and sex, and robust false-negative testing estimates. The optimal policy should combine the strengths of different tests in a hybrid and dynamic manner, adapting to changing pandemic landscapes and public health goals.

Mass AG tests in Israel were used in addition to PCR tests during the Omicron BA.1/BA.2 outbreak, due to limited lab capacities and cost. However, as the number of cases decreases, it is important to reevaluate the use of AG tests. Future surveillance policies must consider the advantages and drawbacks of both PCR and AG tests. It is important to note that the study conducted during the BA.1/BA.2 outbreak is strain-specific, and the results may not be applicable to other strains of the virus.

## Methods

### Data

The Israel Ministry of Health database contains all official AG and PCR tests performed during the Covid-19 pandemic (the database did not contain results of self-AG testing during the study period). We extracted all positive PCR tests conducted between January and April 2022 (a period dominated by the Omicron BA.1/BA.2 variants in Israel) that include information regarding the cycle threshold (Ct) of gene N. Non-integer Ct-values were rounded to their nearest integer values. Data were restricted to tests with Ct ≤ 35, which were considered ‘definitive positive’, and included only PCR tests that had been processed by the five largest Israeli laboratories testing for gene N. These laboratories analyzed samples taken in the community, and did not include tests performed due to hospitalization. For each patient in that dataset, we collected all PCR tests that had a Ct value, as well as all AG test records, using an encrypted patient’s ID number.

The database included the time at which samples were collected for PCR and AG tests, as well as the time at which the PCR positive/negative result was obtained, all provided in a minute time scale. This allowed us to determine whether a patient performed the AG test before or after the PCR test, as well as whether the PCR result was known to the patient at the time the AG test was performed. We considered these factors important in the analysis, as knowledge of one test result can affect the probability of performing the other test. We therefore included in the analysis only individuals who performed an AG test within 24 h after performing a PCR test, using the earliest AG test that was performed within 24 h after the PCR test, but before the PCR result was obtained. To exclude repeated measurements, we used for each individual only the Ct record corresponding to the earliest positive PCR swab sample, among all positive samples taken during January–April 2022.

We also included the following factors as covariates, potentially affecting the probability of obtaining a positive AG result:

1. Vaccination status (In Israel the population was vaccinated almost entirely with the BNT162b2 Pfizer mRNA vaccine), categorized according to the number of doses received before the patient’s PCR sampling date (0, 1, 2, 3, or 4); also included were categories for those who had been infected and for those who had been infected and vaccinated (regardless of the order). As a general rule, individuals belonged to a new vaccination category 7 days after the corresponding vaccination/infection date.
2. Age, using the categorization: 0–2, 3–4, 5–11, 12–15, 16–39, 40–59, and 60 + .
3. Calendar time divided into intervals of 14 days starting from January 1, 2022.
4. Sex.
5. Laboratory (data from the largest five different testing laboratories).

### Statistical analysis

In order to detect any variability that might emerge from different calibration methods and/or different population groups in the different laboratories, we first calculated the distribution of Ct values for each of the labs. We compared these to the distribution in the general population, including patients who did not perform AG tests during the study period. We also compared the distributions of the above-mentioned covariates between the general population and the study population (those performing both PCR and AG tests).

To visualize the relationship between viral load and AG’s detection probability, we used the Ct integer values as categories. For each category, we calculated the proportion of positive AG tests and plotted them, with their corresponding 95% pointwise confidence intervals (CIs), against the Ct value; the latter were obtained using standard intervals for proportions, based on the normal approximation. Similar analyses were conducted for different strata of the covariates. In addition, a logistic line was fitted and plotted using the Ct value as the only continuous covariate, and the AG test’s result as the outcome. In order to examine the effect of the other factors, we fitted a multivariable logistic regression with covariates. Finally, we estimated the proportion of infections that would have remained undetected if all PCR tests had been replaced by AG tests. Specifically, we used the previous multivariable logistic regression results to predict the probability of a negative AG test for all Ct-value records from the 5 laboratories, including individuals who did not take the AG test.

### Ethical approval

The study was approved by an Institutional Review Board (IRB) of the Sheba Medical Center. Helsinki approval number: SMC-8228-21. Due to the retrospective nature of the study, the need of informed consent was waived by Institutional Review Board (IRB) of the Sheba Medical Center.

### Supplementary Information

Supplementary Figures.

## Data Availability

Due to Israel Ministry of Health’s regulations, individual-level data cannot be publicly shared. However, we provide simulated data and code, available at https://github.com/yairgoldy/antigen_ct. Additional requests regarding the data should be referred to amith@gertner.health.gov.il.
