# Supplementary material for: Public health-focused use of COVID-19 rapid antigen and PCR tests
Source: Sci Rep. 2024 Jan 16;14:1430. doi: 10.1038/s41598-023-50533-2 (PMC10792091; doi:10.1038/s41598-023-50533-2)
Supplement: Supplementary file 1 — Supplementary Figures. [file 41598_2023_50533_MOESM1_ESM.docx]

**Supplementary: Public Health-Focused Use of COVID-19 Rapid Antigen and PCR Tests**

Yonatan Woodbridge, Yair Goldberg, Sharon Amit, Naama M. Kopelman, Micha Mandel and Amit Huppert


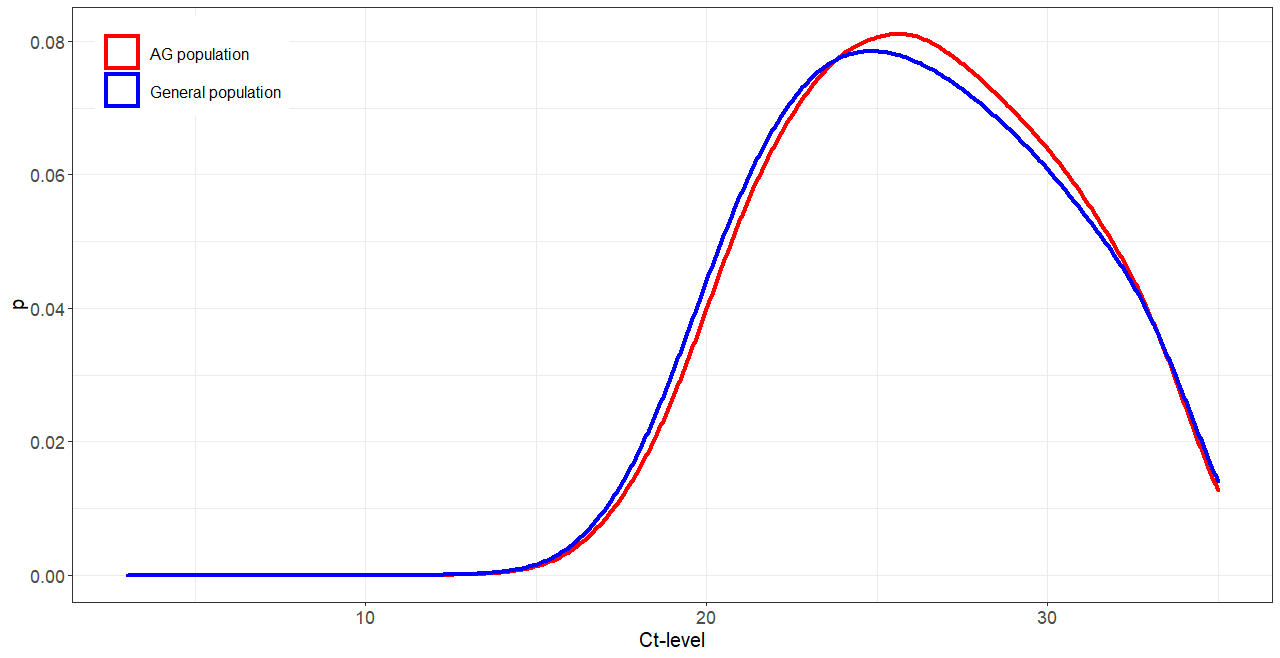


**Fig. S1** Densities of Ct values in the study population (red) and overall population (blue). The two populations are described in Box A (blue curve) and Box C (red curve) of Figure 1.


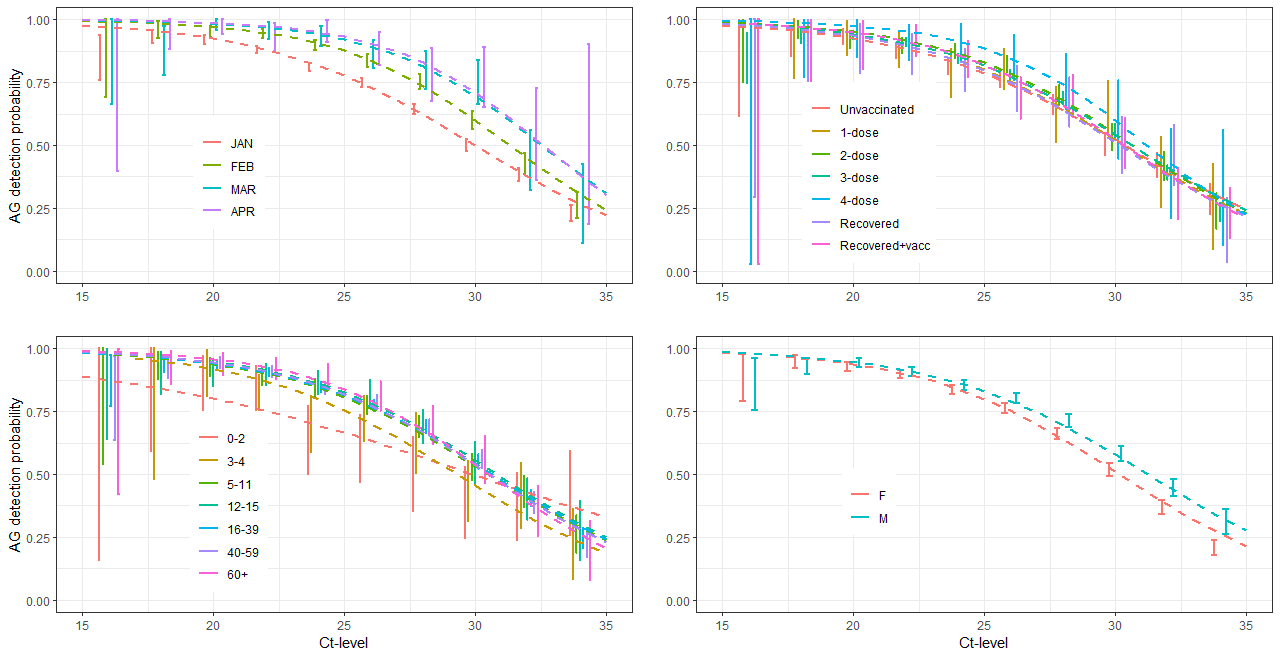


**Fig. S2** The AG detection probability vs Ct-levels for gene N of 5 labs, grouped by 4 different criteria (clockwise from upper left panel): calendar time in months, vaccination status, age group, and sex. Vertical lines are proportion and 95% confidence interval calculated separately for each Ct integer value. The dashed lines are the univariate logistic regressions of Ct value on a positive AG test.


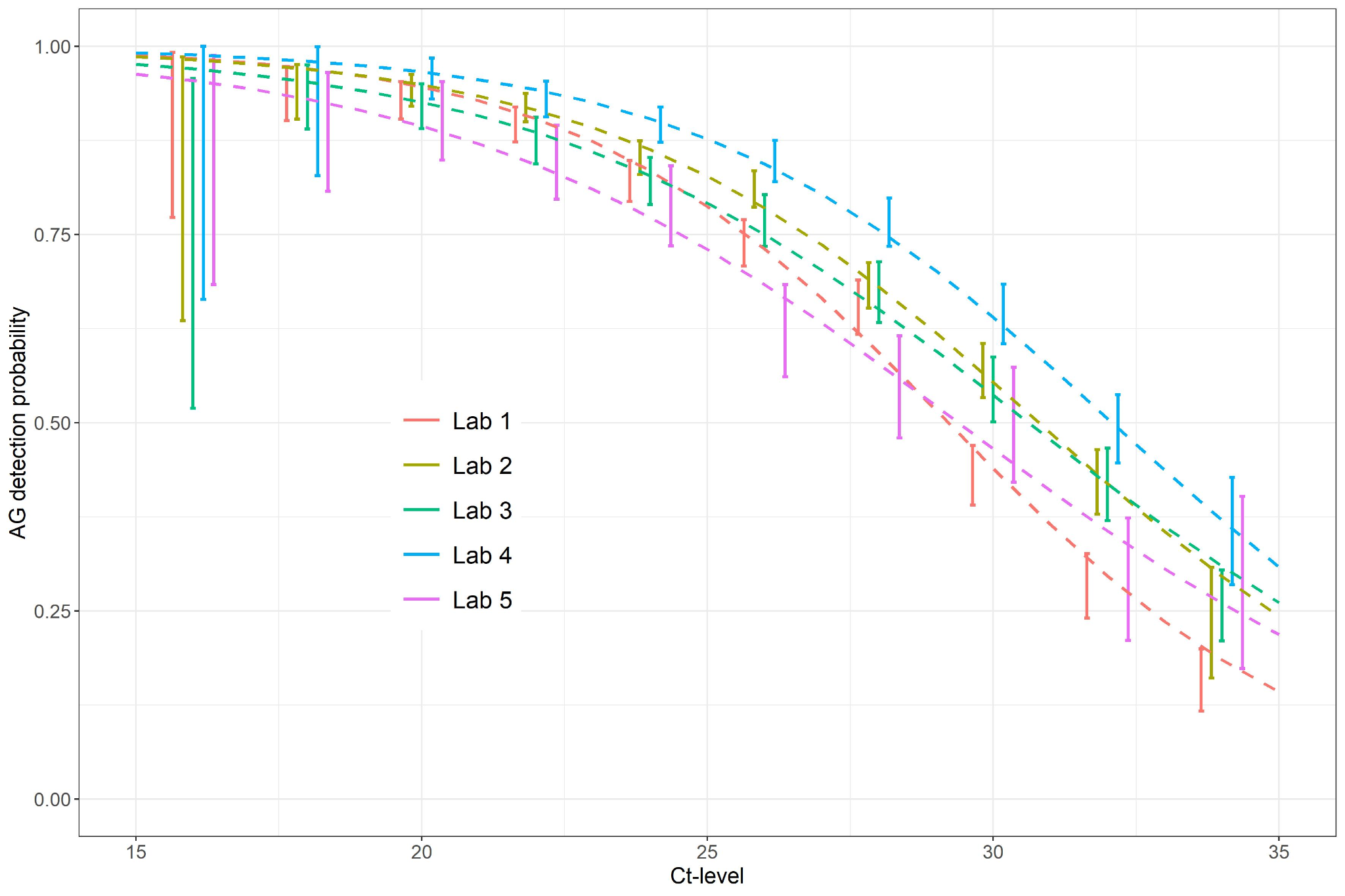


**Fig. S3** The AG detection probability vs Ct-levels for gene N of 5 major labs, grouped by lab. Vertical lines are proportion and 95% confidence interval calculated separately for each Ct integer value. The dashed lines are the univariate logistic regressions of Ct value on a positive AG test.
